# Golgi scaffold protein PAQR3 as a candidate suppressor of gastric cardia adenocarcinoma via regulating TGF‐β/Smad pathway

**DOI:** 10.1002/jcla.24617

**Published:** 2022-07-23

**Authors:** Ying‐Li Wu, Lian‐Lian Hong, Zhe‐Nan Ling, Xuan‐Yu Hu, Zhu Liu, Pei Li, Zhi‐Qiang Ling

**Affiliations:** ^1^ Zhejiang Cancer Institute Cancer Hospital of the University of Chinese Academy of Sciences; Institute of Cancer and Basic Medicine (ICBM), Chinese Academy of Sciences Hangzhou China; ^2^ Department of anaesthesiology Cancer Hospital of the University of Chinese Academy of Sciences; Institute of Cancer and Basic Medicine (ICBM), Chinese Academy of Sciences Hangzhou China; ^3^ Division of Hepatobiliary and Pancreatic Surgery, Department of Surgery, The First Affiliated Hospital Zhejiang University School of Medicine Hangzhou China; ^4^ Department of Pathophysiology School of Basic Medical Sciences Zhengzhou China

**Keywords:** epithelial–mesenchymal transition, gastric cardia adenocarcinoma, PAQR3, prognosis, TGF‐β/Smad signaling pathway

## Abstract

**Objectives:**

To investigate the function of PAQR3 in gastric cardia adenocarcinoma (GCA) and understand the possible mechanism of PAQR3 in regulating epithelial–mesenchymal transition (EMT).

**Methods:**

We detected PAQR3 protein in 146 GCA tissues and paired normal adjacent tissues (PNTs) specimens using immunohistochemical analysis, and explored its clinical significance. The expression levels of PAQR3 protein in 20 GCA tissues, their paired PNTs, HGC27, SGC7901, and GES‐1 cells were analyzed by Western blot. Wild‐type PAQR3 was overexpressed in HGC27 cells. The effects of PAQR3 overexpression on the function of HGC27 cells and its underlying mechanisms were then analyzed through a series of cell and molecular biology experiments.

**Results:**

PAQR3 was significantly down‐regulated in GCA tissues when compared with paired PNTs (*p* < 0.0001). The expression level of PAQR3 in GCA tissues was significantly negatively correlated with *Helicobacter pylori* infection (*p* = 0.000), venous invasion (*p* = 0.000), invasion depth (*p* = 0.000), lymph node metastasis (*p* = 0.022), tumor stage (*p* = 0.000), and patient survival (*p* = 0.009). Downregulation of PAQR3 was highly correlated with increased EMT signature and activated TGF‐β/Smad pathway in GCA tissues. Overexpression of PAQR3 in HGC27 cells negatively regulates its cellular functions, such as cell proliferation and migration, and suppresses EMT. Mechanistically, overexpression of PAQR3 significantly down‐regulates the protein expression levels of TGF‐1, p‐Smad2, and p‐Smad3 in HGC27 cells.

**Conclusion:**

PAQR3 was significantly down‐regulated in GCA tissues, HGC27, and SGC7901 cells. PAQR3 significantly inhibits the proliferation, migration, and invasion of HGC27 cells. Mechanistically, PAQR3 can inhibit the EMT process in HGC27 cells by regulating TGF‐β/Smad signaling pathway.

## Graphical abstract

PAQR3 as a novel tumor suppressor gene can inhibit multiple tumorigenesis and development by regulating multiple signaling pathways, but whether PAQR3 can regulate TGF‐β signaling to influence the progression of GCA is unknown. In this study, we found that PAQR3 expression was lower in GCA tissues, HGC27, and SGC7901 cells. PAQR3 can inhibit the proliferation, migration, and invasion of HGC27 cells. Mechanistically, PAQR3 was able to inhibit the epithelial–mesenchymal transition (EMT) process in HGC27 cells through regulating TGF‐β/Smad signaling.

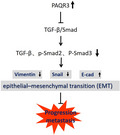

## 1 INTRODUCTION

Gastric cardia adenocarcinoma (GCA) occurring in the cardia location is different from that of distal gastric cancer (GC) because of its certain particularity in the anatomical location and histopathology.^1^ Recently, the incidence of distal GC has begun to decline in many countries and regions, including China, but the incidence of GCA has increasing.^1^, ^2^, ^3^ In the high incidence area of esophageal cancer in China, the incidence of distal GC showed a significant decreasing trend, but the incidence of proximal GC, especially GCA and esophageal–gastric junction adenocarcinoma showed an obvious increasing trend, and the reason is unknown.^1^, ^2^, ^3^ The same change was reflected in terms of tumor mortality. In some areas with a high incidence of esophageal cancer in China, the number of gastric cancer deaths began to rise with the decrease of esophageal cancer deaths, mainly because of the increase in GCA deaths.^3^ Due to the rapid progression and metastasis of GCA, the patient prognosis is poor, with a 5‐year overall survival rate of about 9–25%.^4^, ^5^ Further investigation into the molecular mechanisms underlying GCA carcinogenesis and progression will help to improve cure rates.

Transforming growth factor beta (TGF‐β) is one of the members of the transforming growth factor family with a molecular weight of about 25KD, a polypeptide cytokinine with multiple biological activities, which can be involved in the regulation of various biological behaviors, either by autocrine or paracrine means, including cell growth, differentiation, apoptosis, adhesion, and angiogenesis.^6^ The classical TGF‐β pathway is mediated through the Smads protein family, and its main member is Smad (drosophila mothers against decapentaplegic protein)2/3/4.^6^ Smads proteins, an important member of the TGF‐β pathway and a critical step in transducing TGF‐β signaling from extracellular to within the nucleus.^6^ In addition, TGF‐β can also activate multiple signaling pathways within cells to regulate cellular biological behavior, such as Ras (Ras protein)/MAPK (mitogen‐activated protein kinase)/ERK (extracellular regulated MAP kinase), JNK (c‐Jun N‐terminal kinases)/P38 (p38 kinase), PI3K (phosphatidylinositol 3‐kinase)/AKT (AKT serine/threonine kinase), RohA (RohA kinase), PP2A (protein phosphatase 2)/p70s6K (ribosomal protein S6 kinase beta‐1) pathway, etc.^6^, ^7^, ^8^ Abnormal activation of the TGF‐β pathway is one of the most commonly altered signaling pathways in tumors, which has been reported to play a key role in multiple tumorigenesis and development.^9^

The PAQR3 (progestin and adipoQ receptor family member 3) gene is located on chromosome 4q^21^ and encodes a 37 kDa membrane protein that is highly conserved. The N terminus of PAQR3 is toward the cytoplasm and the C terminus in the Golgi apparatus, which belongs to the third member of the PAQR superfamily.^10^, ^11^ PAQR3 is a recently identified gene with a variety of potential tumor suppressor functions. PAQR3 binds the B‐Raf (B‐Raf proto‐oncogene, serine/threonine kinase), C‐Raf (C‐Raf proto‐oncogene, serine/threonine kinase) within the cytoplasm and sequesters it to Golgi apparatus, interfering with the binding of the Raf kinase to its upstream and downstream signaling molecules, and inhibiting Ras/Raf/MEK (mitogen‐activated protein kinase kinase 1)/ERK pathway.^10^, ^11^ PAQR3 knockout mice, with the loss of the inhibition of Raf by PAQR3, significantly promoted skin cancer induced by dimethylbenanthracene/fobol (DMBA/TPA).^12^ PAQR3 inhibits ERK activation, proliferation, and tumorigenicity of human malignant melanoma cell line A375 cells.^13^ PAQR3 is able to interact with Gβγ to inhibit PI3K/AKT signaling downstream of the G protein‐coupled receptors.^14^ PAQR3 inhibits the formation of HIF‐1a (hypoxia inducible factor 1 subunit alpha) /p300 (E1A‐binding protein p300) complexes by inhibiting MAPK signaling, thereby endothelial growth, migration and angiogenesis, and signaling downstream of VEGF (vascular endothelial growth factor).^15^

In functional and mechanistic studies involving PAQR3 inhibition of tumor metastasis, it has been found that simultaneous loss of PAQR3 and p53 (p53 tumor suppressor) can accelerate spontaneous skin‐like tumor formation and promote EMT (epithelial–mesenchymal transition) tumorigenesis.^16^ Knockdown of PAQR3 expression in the colon cancer cell line SW‐480 cells significantly promoted cell proliferation, enhanced EGF (epidermal growth factor)‐stimulated ERK phosphorylation and β‐catenin entering into the nucleus, and knockdown in Apc (APC regulator of WNT signaling pathway)^Min/+^ mice aggravated intestinal carcinogenesis in mice.^17^ PAQR3 has recently been found to be a key protein involved in GC progression, and its reduced expression levels are correlated with enhanced EMT phenotype characteristics in GC cells. Overexpression of PAQR3 in AGS cells significantly inhibited the cell growth, migration, and EMT process, with a negative regulation of GC cells. Therefore, PAQR3 is considered as a novel and important gene with multiple tumor suppressor functions in GC.^18^ PAQR3, as a newly identified gene with a potential tumor suppressor function, as a novel tumor suppressor gene, can inhibit multiple tumorigenesis and development by regulating multiple signaling pathways,^19^ however, whether PAQR3 can modulate the TGF‐β pathway to influence GCA progression remains unknown. Here, we explored the role of PAQR3 in GCA and elucidated the underlying mechanism by which PAQR3 regulates the TGF‐β/Smad pathway to inhibit the EMT.

## 2 MATERIALS AND METHODS

### 2.1 GCA specimens, cell lines

GES‐1 (a human fetal normal gastric mucosa epithelial cell line), HGC27, and SGC7901 (two GC cell lines) were purchased from Shanghai Cell Bank, Chinese Academy of Sciences. Cells were cultured in Gibco RPMI 1640 containing 10% heat‐inactivated fetal bovine serum (FBS) (Gibco, Invitrogen), penicillin and streptomycin (both concentrations: 100 U/ml) (Sigma‐ Aldrich, Inc.), maintained at a CO_2_ Incubator, requiring a stable temperature (37°C), stable CO_2_ levels (5%), constant pH (pH:7.2–7.4), and higher relative saturation humidity (95%). This study included 146 GCA patients who underwent surgical treatment at Zhejiang Cancer Hospital (Hangzhou China) from January 2015 to December 2019, and patients did not receive any preoperative treatment. All patients were diagnosed independently with pathological histology by two professional clinicopathologists. The tumor stage was performed according to the criteria for the international TNM (tumor‐node‐metastasis) staging system established by the American Joint Joint Committee on Cancer (AJCC, American Joint Committee on Cancer) and the International Alliance Against Cancer (UICC, Union for International Cancer Control). The clinicopathological information of all patients was collected and the detailed clinicopathological information was shown in Table 1. The study was approved by the Institutional Review Board of Zhejiang Cancer Hospital (Ethical Certification No. zjzlyy [2015]‐02–125, and zjzlyy‐IRB‐2019–98), and patient informed consent was obtained for the use of all tissue specimens. All patients received postoperative adjuvant chemotherapy with S‐1. The prognosis of GCA patients were assessed by their overall survival (OS), defined as the time from surgery to death. Therefore, all enrolled GCA patients were followed up until the due date.

**TABLE 1:** Correlation analysis of PAQR3 protein expression levels and clinicopathological parameters in GCA patients

Variables	*N*	PAQR3 protein level		*χ* ^2^	*p*‐Value
		Absent/Low, *n* (%)	normal/High, *n* (%)		
Gender					
Male	108	67	41	0.015	1.000
Female	38	24	14		
Age at diagnosis					
<60	73	46	27	0.029	1.000
≥60	73	45	28		
Lauren classification					
Intestinal	120	73	47	0.257	0.808
Diffuse	21	14	7		
*Helicobacter pylori*					
Negative	48	17	31	22.058	**0.000**
Positive	98	74	24		
Tumor size (cm)					
<5	57	35	22	0.034	0.863
≥5	56	33	89		
Differentiation					
High/medium	73	48	25	0.729	0.495
Low	73	43	30		
Venous invasion					
No	87	44	43	12.668	**0.000**
Yes	59	47	12		
Nerve invasion					
No	51	33	18	0.189	0.722
Yes	95	58	37		
Invasive depth					
T1/T2	31	7	24	26.482	**0.000**
T3/T4	115	84	31		
Nodal metastasis					
No	41	19	22	6.206	**0.022**
Yes	105	72	33		
Distant metastasis					
No	129	78	51	1.639	0.288
Yes	17	13	4		
TNM stage					
I/II	55	24	31	13.131	**0.000**
III/IV	91	67	24		

*Note*: Chi‐square test was used for statistical analyses. *p* < 0.05 is considered statistically significant. PAQR3 protein lower expression is defined as IHC score ≤4. Five cases were not included in statistical analysis for tissue type, including three squamous cancers, one neuroendocrine tumor, and one mesostima. Bold values indicates statistically significant differences.

### 2.2 Immunohistochemical staining

Immunohistochemical (IHC) staining on tissue sections was carried out by an ABC method (Avidin–Biotin) and observed by 3,30‐diaminobenzidine tetrahydrochloride. The following antibodies were used for IHC staining: anti‐PAQR3 antibody and anti‐Snail antibody (Abgent Inc., 9765 Clairemont Mesa Blvd Suite C San Diego, CA 92124, ), anti‐TGF‐β antibody, anti‐Snail antibody, anti‐pSmad2 antibody, anti‐E‐cadherin antibody, and anti‐pSmad3 antibody (CST‐US subsidiary ). The IHC results were assessed as previously reported by semi‐quantitative scoring methods.^20^, ^21^

### 2.3 Western blot analysis

Western blot (WB) was performed as the standard protocol.^22^, ^23^ Reagents and antibodies used for immunoblotting analysis are as described below. Radio Immunoprecipitation Assay (RIPA) Lysis buffer, Enhanced BCA Protein Assay Kit (Shanghai BiYuntian Biotechnology Co., Ltd., Songjiang Science and Technology Entrepreneurship Center, Lane 1500); polyvinylidene fluoride (PVDF) membrane (290 Concord Rd., Billerica); Tris‐buffered saline and Tween (TBST), Tween‐20 (Beijing Solaibao Technology Co., LTD. ); anti‐PAQR3 antibody and anti‐Snail antibody (Abgent Inc.); anti‐E‐cadherin antibody, anti‐TGF‐β1 antibody, anti‐pSmad2 antibody and anti‐pSmad3 antibody (CST‐US subsidiary); affinity‐purified goat anti‐rabbit IgG antibody (Sigma‐Aldrich, Inc.); β‐actin (Santa Cruz Biotechnology [Shanghai] Co., Ltd.).

### 2.4 Materials and methods related to cell‐based experiments

The Myc (***MYC*** proto‐oncogene)‐tagged PAQR3 for overexpression experiments was performed as described previously.^10^ A series of cell biology experiments, including the colony formation test, the MTT (3‐[4,5‐ Dimethylthiazol‐2‐yl]‐2,5‐diphenyltetrazolium bromide) test, and the wound healing test, were carried out as previously described.^15^, ^17^ Total RNA of cultured cells was isolated by TRIzol kit (Thermo Fisher Technology [China] Co., LTD.) according to the instructions. Reverse transcription of the mRNA was performed by the PrimeScript RT kit (Takara Biomedical Technology [Beijing] Co., Ltd.). The abundance of PAQR3 mRNA expression in cultured cells was detected by fluorescence quantitative RT‐PCR (reverse transcription polymerase chain reaction). The primer sequences for PAQR3 were shown as follows: forward‐5′‐TTCAGCAAGTGCGTCCAGAG‐3′; reverse‐5′‐TGCCATCCATCTTCGACAT G‐3′. The primer sequences for β‐actin were shown as follows: forward‐5′‐GATCATTGCTCCTCCTGAGC; reverse‐5′‐ACTCCTGCTTGCTGATCCAC‐ 3′. Quantitative RT‐PCR reactions were performed on the ABI7500 Rapid PCR Instrument (Thermo Fisher Technology [China] Co., LTD.) using Takara Bio's SYBR® Premix Ex Taq (Tli RNase H Plus) (Takara Biomedical Technology [Beijing] Co., Ltd.), strictly following standard quantitative PCR procedures. All experiments were required to be repeated three times, and the median was taken as the result of this experiment.

### 2.5 Statistical analysis

All data were analyzed by IBM SPSS Statistics version 23.0 (SPSS Inc.). A paired t‐test for normal distribution by Fisher's exact test was used for the analysis of differences between groups. The Kaplan–Meier method was used for the survival analysis of GCA patients, and the log‐rank test was used for assessing their significance. A *p* value < 0.05 was determined as statistically significant.

## 3 RESULTS

### 3.1 PAQR3 expression was significantly downregulated in GCA tissues and GC cell lines when compared with normal tissues and normal cell lines

**FIGURE 1:**
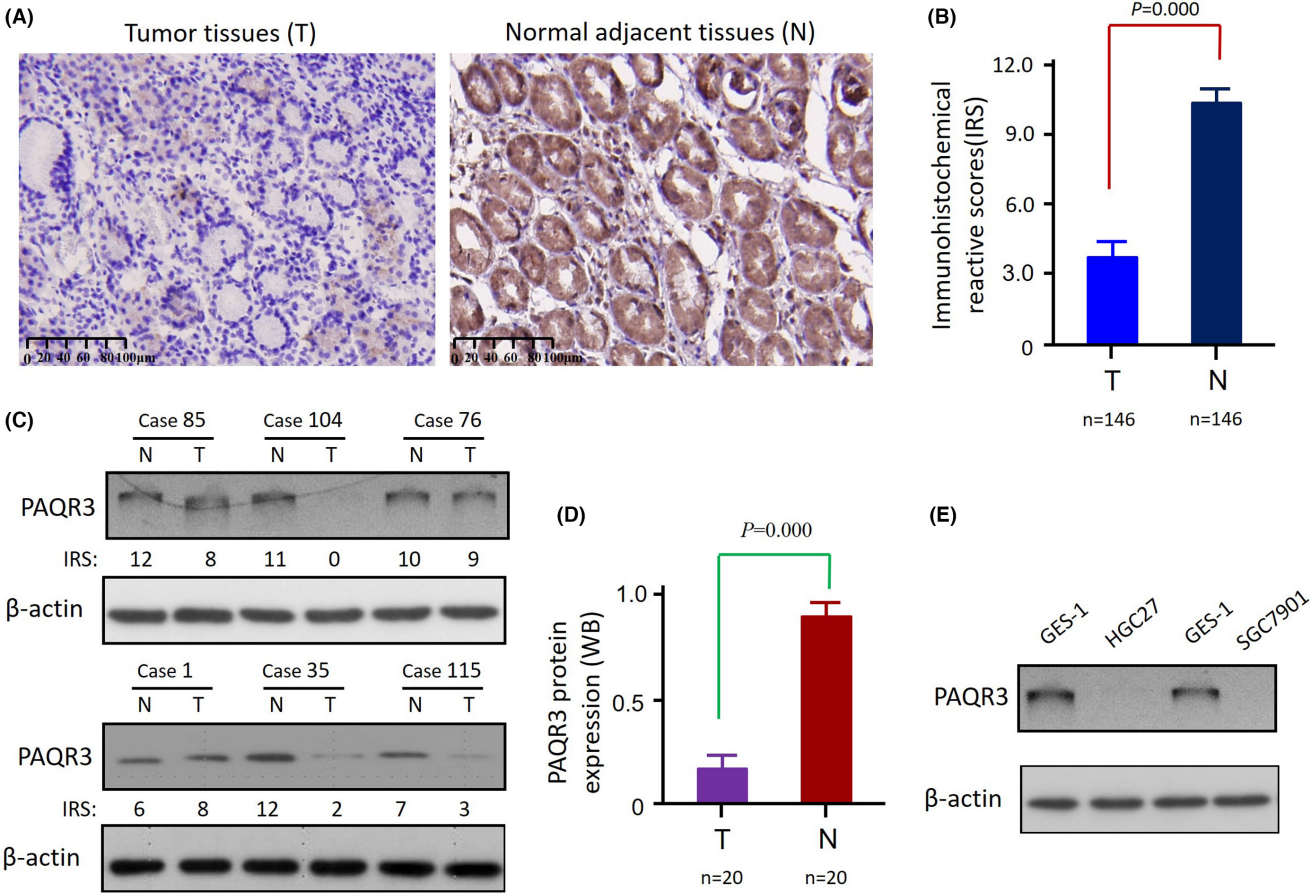
PAQR3 expression was significantly down‐regulated in primary GCA tissues. (A) Representative immunohistochemical images of PAQR3 with low expression in GCA tissues and high expression in their paired cancerous adjacent normal tissues. 200× for all images. (B) IHC results were scored using a 0–12 semi‐quantitative scoring system, and compared with their paired cancerous adjacent normal tissues, PAQR3 expression was significantly downregulated in GCA tissues, with a statistically significant difference. (*p* = 0.000). (C) The expression levels of PAQR3 in GCA tissues and their paired adjacent normal tissues were analyzed by Western blot (WB). PAQR3 protein detected by WB analysis was consistent with that detected by immunohistochemical analysis. IRS: immunohistochemical reactive scores; N: normal adjacent tissues; T: tumor tissues. (D) The WB results showed that PAQR3 was significantly downregulated in GCA tissues compared to their paired cancerous adjacent normal tissues, with a statistically significant difference (*p* = 0.000). (E) WB analysis of PAQR3 protein expression in GES‐1, HGC27, and SGC7901 cells.

The levels of PAQR3 protein expression in 146 GCA tissues and their paired PNTs were detected by immunohistochemical (IHC) staining, results showed PAQR3 down‐regulation was detected in 62.3% (91/146) GCA tissues, while PAQR3 in PNTs, 1.4% (2/146) cases were expressed at low levels and 98.6% (144/146) at normal/high levels, the difference between them was significant (*p* = 0.000) (Figure 1A,B). To further validate the IHC results, using clinical specimens to verify PAQR3 expression in GCA tissues and paired PNTs by Western blot, a reduced PAQR3 expression in GCA tissues compared with 20 paired PNTs was observed, and the results were statistically significant (*p* = 0.000) (Figure 1C,D). The same finding was obtained in GC cell lines, where PAQR3 protein expression was significantly downregulated in HGC27 and SGC7901 cells when compared with the human gastric epithelial cell line GES‐1 cells (Figure 1E).

To investigate the significance of PAQR3 downregulation in GCA tissues, we explored the correlation between PAQR3 protein expression levels and clinicopathological parameters in GCA patients (Table 1). The results showed that PAQR3 protein expression levels in GCA tissues were significantly negatively associated with *Helicobacter pylori* (*H. pylori*) infection (*p* = 0.000), venous infiltration (*p* = 0.000), depth of infiltration (*p* = 0.000), etc. However, PAQR3 protein expression levels were not associated with GCA patient sex (*p* = 1.000), age at diagnosis (*p* = 1.000), Lauren classification (*p* = 0.808), etc. Together, these results indicate that PAQR3 protein levels are significantly downregulated in GCA tissues and are closely associated with the tumor malignant phenotype, suggesting that PAQR3 plays an important role in GCA.

### 3.2 Effect of PAQR3 protein expression levels in tumor tissues on the prognosis of GCA patients

**FIGURE 2:**
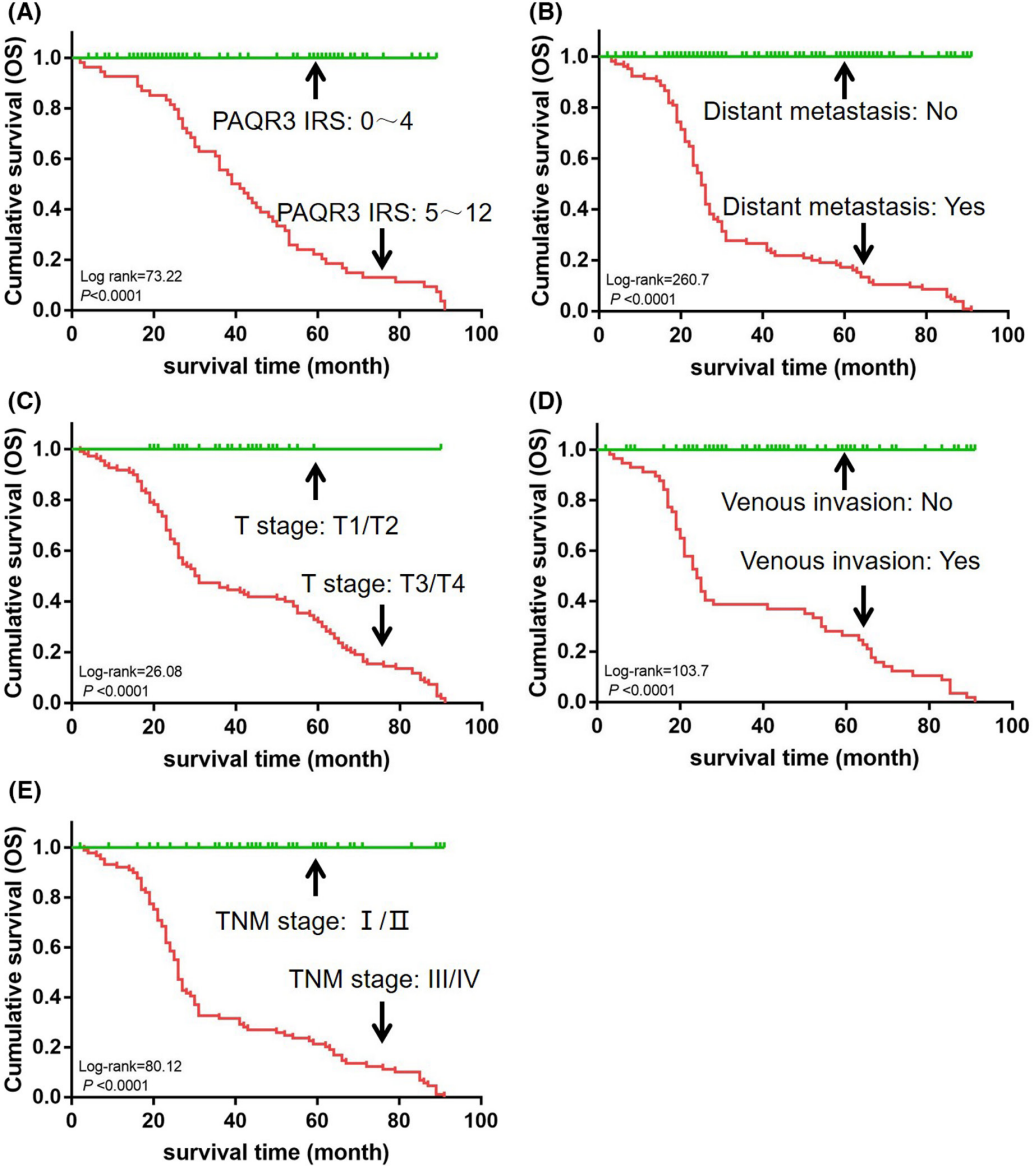
The survival analysis of GCA patients based on PAQR3 expression level and clinicopathological characteristics. (A) The Kaplan*–*Meier (K‐M) overall survival (OS) curves in GCA patients who have been treated with primary gastrectomy based on PAQR3 expression level. (B–E) OS in GCA patients based on different clinicopathological characteristics

We explored the effect of PAQR3 protein expression levels in tumor tissues on the overall survival (OS) of GCA patients. Survival analysis showed that the mean OS (IHC score = 0–4) of GCA patients with reduced PAQR3 protein expression was 32.8 months, and the mean OS of GCA patients with normal PAQR3 expression or elevated PAQR3 expression was 49.6 months, with a significant difference (*p* < 0.05) (Figure 2). However, the correlation between the PAQR3 expression levels in the tumor tissues and the survival of the GCA patients depends on the tumor stage. Downregulation of PAQR3 expression was closely related with tumor progression. Besides the level of PAQR3 expression in tumor tissues affects the prognosis of GCA patients, these clinicopathological features, including distant metastasis, T stage, venous infiltration, and TNM stage, all have significant effects on the prognosis of GCA patients (Figures 2B–E).

### 3.3 Downregulation of PAQR3 expression in GCA tissues is highly correlated with increased EMT phenotypic features and activated TGF‐β/Smad pathway

**FIGURE 3:**
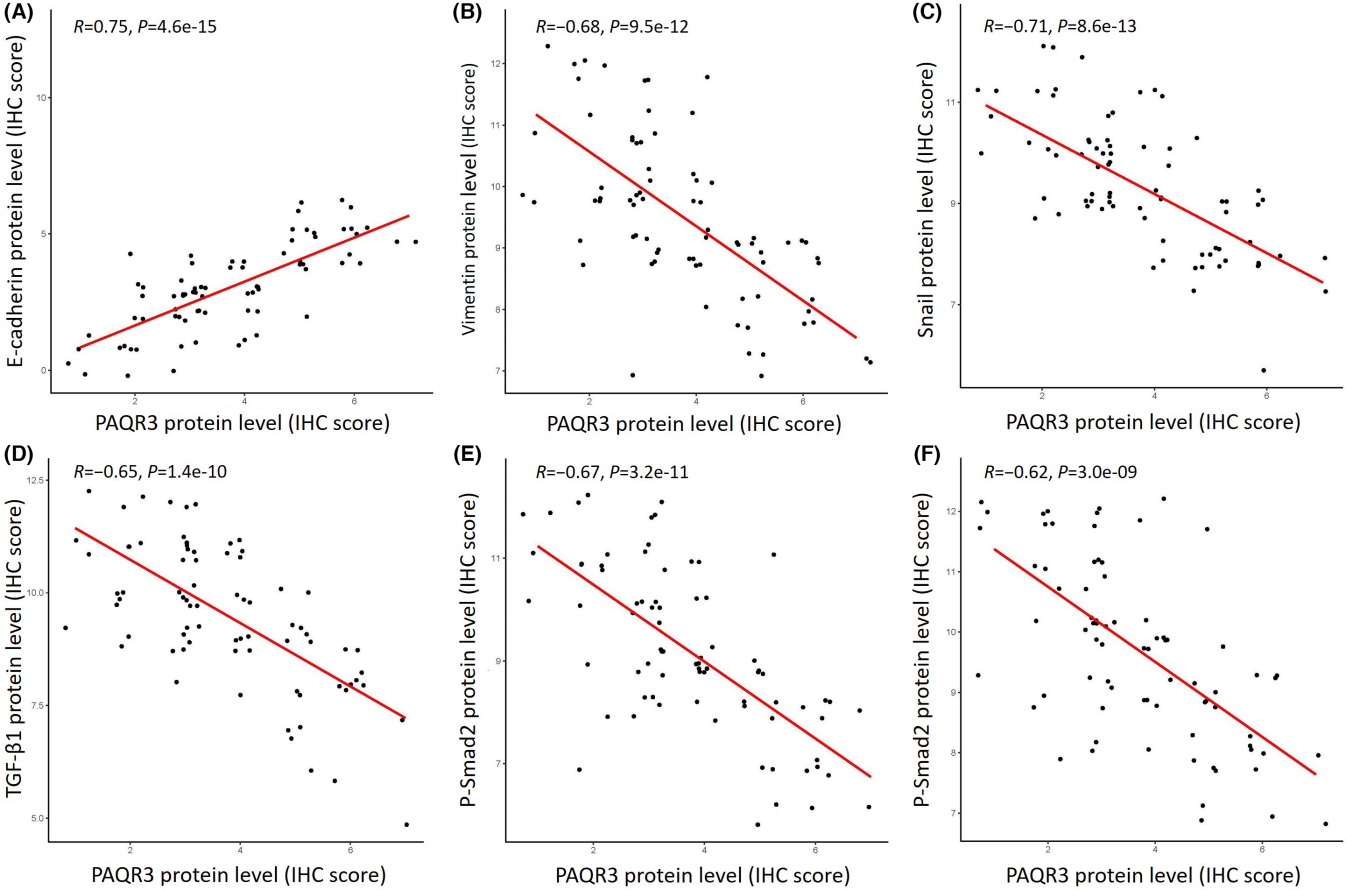
Correlation analysis between PAQR3 protein expression levels and epithelial–mesenchymal transition (EMT) markers or TGF‐β/Smad signaling pathway activation. The protein levels of PAQR3, e‐cadherin, vimentin, Snail, TGF‐1, p‐Smad2, and p‐Smad3 were analyzed in GCA tissues by immunohistochemical staining. The immunohistochemical scores of PAQR3 in GCA tissues were plotted with those of EMT‐related markers or with key proteins of the TGF‐β/Smad signaling pathway. A significant positive relationship was found between PAQR3 protein expression in GCA tissues and E‐cadherin protein expression (A), but a significant negative relationship was found between PAQR3 protein expression in GCA tissues and Vimentin protein expression (B), or Snail protein expression (C), or TGF‐β1 protein expression (D), or p‐Smad2 protein expression (E), or p‐Smad3 protein expression (F).

To investigate the correlation of PAQR3 expression and GCA metastasis mediated by the TGF‐β/Smad signaling pathway, we used IHC to examine the expression levels of EMT‐related markers, including E‐cadherins, Vimentin, Snail proteins, and TGF‐β/Smad signaling pathway‐related proteins in GCA tissues, and explored the correlation of PAQR3 with the expression of these proteins. Correlation analysis based on IHC score found a significant positive correlation between PAQR3 protein expression and E‐cadherin expression in GCA tissues (*R* = 0.75, *p* < 0.0001) (Figure 3A). Meanwhile, PAQR3 protein expression in GCA tissues was significantly negatively correlated with Vimentin expression (*R* = −0.68, *p* < 0.0001) (Figure 3B), Snail expression (*R* = −0.71, *p* < 0.0001) (Figure 3C), TGF‐β1 expression (*R* = −0.65, *p* < 0.0001) (Figure 3D), p‐Smad2 expression (*R* = −0.67, *p* < 0.0001) (Figure 3E), and p‐Smad3 expression (*R* = −0.62, *p* < 0.0001) (Figure 3F). Thus, our results suggest that the downregulation of PAQR3 in GCA tissues promotes tumor progression and metastasis, which is closely related to activation of TGF‐β/Smad signaling, subsequently mediating increased EMT phenotype characteristics.

### 3.4 Effect of PAQR3 overexpression on cell proliferation, migration, activation of TGF‐β/Smad pathway, and EMT phenotypic characteristics of HGC27 cells

**FIGURE 4:**
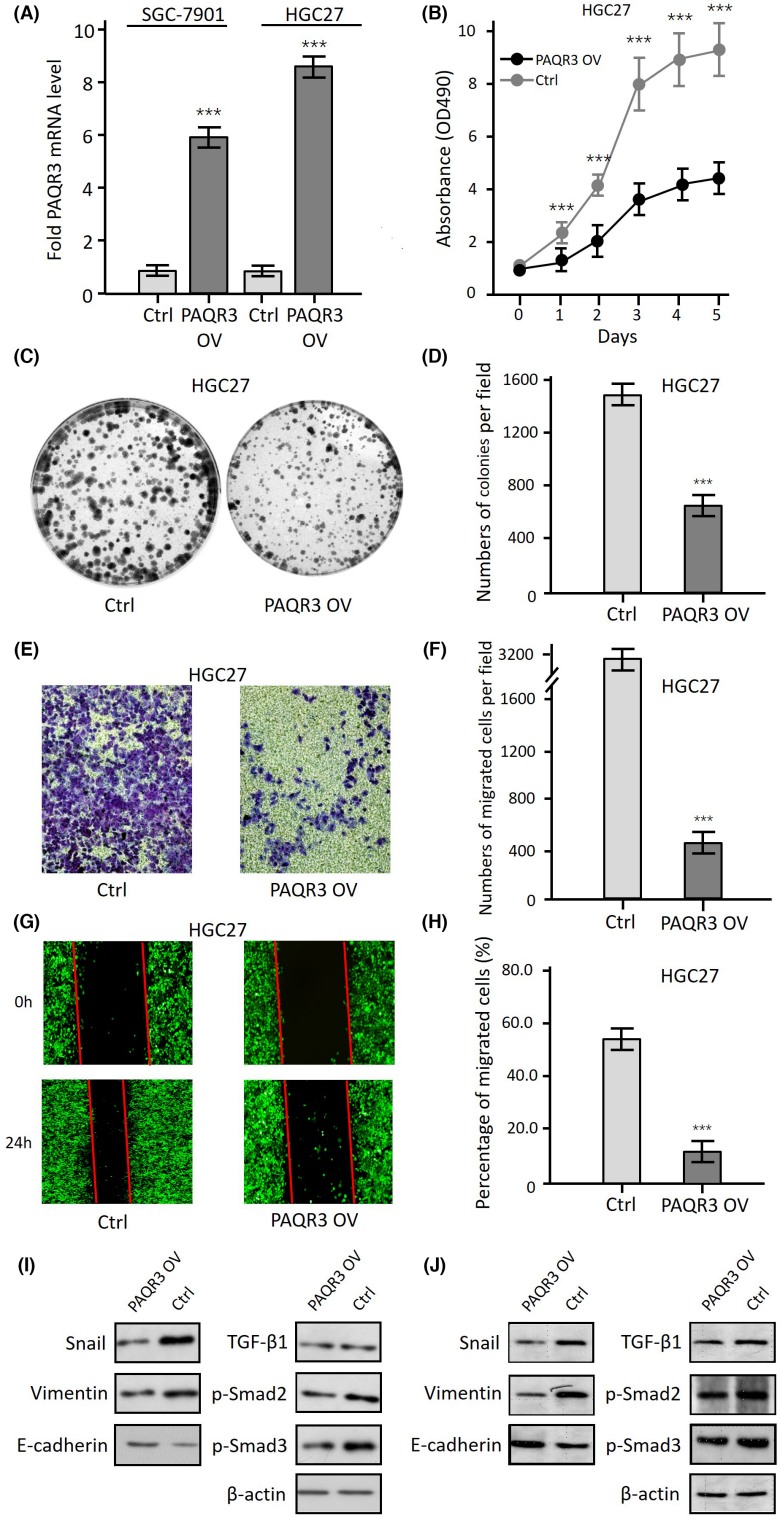
Regulation of cell proliferation, migration, TGF‐β/Smad signaling and EMT‐related gene expression in HGC27 cells by PAQR3. (A) The expression abundance of PAQR3 mRNA in SGC7901 cells with PAQR3 overexpressing, HGC27 cells with PAQR3 overexpressing, and their controls was determined by quantitative RT‐PCR, respectively. Data were presented as the mean ± standard deviation (SD). ***p < 0.001. (B) The rates of cells growth was determined by MTT analytical method at indicated time point using HGC27 cells overexpressing PAQR3 (PAQR3 OV) and HGC27 cells with an empty vector (Ctrl). Data were presented as the mean ± standard deviation (SD). ***p < 0.001. (C) Colony formation experiments were performed as follows: HGC27 cells in the log‐growth phase were seeded in six wells, with 400 cells per well, and clones were counted after 5 days of culture. (D) Statistical analysis results was shown. Data were presented as the mean ± standard deviation (SD). ***P < 0.001. (E) Transwell experiments were performed as follow: HGC27 cells in the log‐growth phase were seeded in a 24‐well transwell chamber, and cells in the lower chamber were fixed after 24 h of culture, then cells were counterstained with crystal violet. (F) The results of the statistical analysis were shown. Data were presented as the mean ± standard deviation (SD). ***p < 0.001. (G) The scratch wound healing experiment was carried out as follows: HGC27 cells were transfected overexpressing PAQR3 plasmid or with a control plasmid, and then photographed at 0 and 24 h after scratch to analyze the effects of PAQR3 overexpression on cell migration. (H) The results of the statistical analysis were shown. Data were presented as the mean ± standard deviation (SD). ***p < 0.001. (I and J) HGC27 cells were transfected with a control plasmid or overexpressed PAQR3 plasmid, and after 3 days of culture, the effects of PAQR3 on the expression of EMT‐related genes and the expression TGF‐β/Smad signaling key protein were detected by immunoblotting with antibodies. I:first WB results, J: second WB results for the same sample.

To explore the role of the PAQR3 gene in GC cells in vitro, cell lines with Myc‐tagged PAQR3 for overexpression were successfully constructed. PAQR3 mRNA expression levels were 8.7 times higher in HGC27 cells overexpressing PAQR3 compared to its control cells, and 5.9 times higher in SGC7901 cells overexpressing PAQR3 than its control cells (Figure 4A). Thus, HGC27 cells overexpressing PAQR3 were selected for a subsequent series of cell function and molecular mechanistic experiments. We found by MTT that overexpression of PAQR3 significantly inhibited cell proliferation (Figure 4B) and reduced colony formation in HCG27 cells (Figure 4C,D). The Transwell analysis revealed that the overexpression of PAQR3 in HGC27 cells had significant inhibitory effects on cell migration (Figure 4E,F) and scratch healing (Figure 4G,H). Mechanistically, overexpression of PAQR3 in HGC27 cells could inhibit the EMT process mediated by the TGF‐β/Smad signaling pathway, and overexpression of PAQR3 in HGC27 cells upregulated the expression level of E‐cadherin and downregulated the expression levels of Snail and Vimentin (Figure 4I,J).

In conclusion, in vitro cell function assays showed that PAQR3 functions as a tumor suppressor gene in HGC27 cells, significantly inhibiting the proliferation, migration, EMT process and activation of TGF‐β/Smad signaling pathway in HGC27 cells. This is consistent with our clinical findings, where PAQR3 was significantly downregulated in GCA tissues when compared with their adjacent normal tissues, and showed a significant negative correlation with the progression and metastasis of GCA.

## 4 DISCUSSION

Previous studies showed that PAQR3 is a newly discovered gene with a potential tumor suppressor function in a variety of tumors, with low expression in liver cancer, colorectal cancers, gastric cancer, *etc*., and significantly negatively correlated with patient tumor progression and poor prognosis.^16^, ^17^, ^18^, ^19^ Here, we mainly investigated the role of PAQR3 in GCA progression, demonstrated that PAQR3 is low expressed in GCA tissues and PAQR3 has an inhibitory function on the proliferation, migration, and invasion of HCG27 cells in vitro; further clarified that PAQR3 can inhibit the EMT process by inhibiting the activation of TGF‐β/Smad signaling pathway.

Epithelial cell–mesenchymal transition refers to the biological process in which epithelial cells become cells with a stromal phenotype through specific programs, characterized by loss of cell adhesion and enhanced cell motility. In the process of EMT, the expression of cell adhesion molecules (such as E‐cadherin) is reduced, the cytokeratin cytoskeleton is converted into vimentin (Vimentin)‐based cytoskeleton, and its morphology has the characteristics of mesenchymal cells. Through EMT, epithelial cells lose cell polarity and lose epithelial phenotypes such as junctions to the basement membrane, and acquire high stromal phenotypes such as migration and invasion, anti‐apoptosis and the ability to degrade the extracellular matrix. EMT is an important biological process by which epithelial cell‐derived malignant tumor cells acquire migration and invasion capacity.^24^ Jiang et al.^16^ found the synergistic function of PAQR3 and p53 in tumorigenesis, also found that they synergistically participate in the EMT process of tumor cells, thus proposed that p53 is a “checkpoint” regulating EMT, and only tumor cells losing the “checkpoint” can effectively perform EMT and then participate in tumor infiltration and metastasis. Therefore, this study firstly identified the involvement of PAQR3 in the function of EMT. In present study, using the HGC27 cell model, it was found that PAQR3 overexpression could upregulate E‐cadherin protein expression, downregulate Snail and Vimentin expression, suggesting the function of PAQR3 in promoting EMT in HGC27 cells.

As an important factor regulating tumor progression and metastasis, EMT is driven by the activation of various signaling pathways such as AKT/mTOR, Wnt/β‐catenin, notch, TGF‐β/Smad, etc. TGF‐β is a key signaling molecule regulating EMT.^24^ TGF‐β is a group of newly identified TGF‐superfamilies that regulate cell growth and differentiation. In recent years, TGF‐β has been found to have important regulatory effects on cell growth, differentiation and immune function, such as inhibition of epithelial cell and endothelial cell growth, inhibition of lymphocyte differentiation and inhibition of immunoactive cell proliferation. Clearly, these biological functions can inhibit the development of tumor cells.^25^, ^26^ However, it has been shown that TGF‐1 can also promote the infiltration and metastasis of tumor cells while regulating the cellular immune system and the microenvironment of tumor tissues.^26^, ^27^ The effect of TGF‐β1 is pleiotropic, and almost all cells in the human body can be synthesized and secreted. TGF‐1 plays a key role in its regulation during cell growth and differentiation, extracellular matrix formation, immune regulation, angiogenesis, apoptosis as well as tumor development and progression.^28^, ^29^ In TGF‐β/Smad signaling, the TGF‐1 kinase first phosphorylates the carbon‐end residues of Smad2 and Smad3 proteins, further forms a complex with Smad4 protein, and then the nuclear translocation of the complex regulates downstream gene expression, thereby stimulating the development of EMT.^30^, ^31^ In this study, we examined protein changes associated with TGF‐β/Smad pathway and showed that TGF‐beta1, p‐Smad2 and p‐Smad3 expression decreased after overexpression of PAQR3 in HGC27 cells. Therefore, we hypothesized that PAQR3 can inhibit the EMT process in GC cells by inhibiting TGF‐β/Smad signaling.

## AUTHOR CONTRIBUTIONS

Wu YL, Hong LL, Ling ZN, Hu XY, Liu Z, Li P and Ling ZQ participated in the design of the study, experimental execution, data analysis, and manuscript discussion throughout the process. Wu YL, Hong LL, Ling ZN, Hu XY and Liu Z performed the experiments. Wu YL drafted the manuscript. LING ZQ was responsible for manuscript revision and Li P made constructive comments on the manuscript. Hong LL, Ling ZN, and Hu XY and Liu Z provided very important help for the analysis and validation of the experimental data. In the initial manuscript and subsequent revisions, all authors contributed to the discussion and suggested very valuable revisions to the manuscript. All authors read the final version of the manuscript and agreed to submission.

## FUNDING INFORMATION

The scientific research projects funding the study are as follows: National Natural Science Foundation of China (81972908), National Health Commission Science Research Fund‐Zhejiang Provincial Health Key Science and Technology Plan Project (WKJ‐ZJ‐2117), Key Projects from Zhejiang Provincial Natural Science Foundation (LZ18H160002), General Project from Zhejiang Provincial Natural Science Foundation (LQ19H160005), Leading Talents in Scientific and Technological Innovation from Zhejiang Provincial Ten Thousand Talents Plan (Zhejiang Provincial CPC Committee Talents [2019]‐3), Zhejiang Province Health Leader Talent (Zjwjw2021‐40), and Major Training Personnel from Zhejiang Provincial Program for Training and Development Project for 151 Talents (Zjhrss2014‐150). Program for Innovation Research Team (in Science and Technology) in University of Henan Province (Grant No. 20IRTSTHN026). Zhejiang Medical and Health Science and Technology Project (2019ZD025). Zhejiang Provincial Public Welfare Technology Research Plan Project (LGD20H160003).

## CONFLICT OF INTEREST

There were no potential competing financial or other interests in the study.

## Data Availability

Raw experimental data used to support the findings of this paper are available from the corresponding author on reasonable request.
